# The Story of the Insane from Year to Year

**Published:** 1904-10-22

**Authors:** 


### THE STORY OF THE INSANE FROM YEAR TO YEAR.

Seventeenth Annual Series.

CITY OF LONDON ASYLUM AT DARTFORD.

On January 1st there were 542 patients in residence, and on December 31st the number had risen to 563. The first admissions reached a total of 184, the readmissions 9, the recoveries 59, the relieved 62, and the deaths 48. The total number under treatment during the year was 735, and the average number resident was 561. Calculated on the admissions the recoveries stand at 43 70 per cent., and calculated on the average number resident the deaths stand at 8·56; the former being decidedly above the average and the latter a little under it. Of the year's deaths 18 were caused by cerebral and spinal diseases (general paralysis accounting for 11 of these), 6 by consumption, and there was no death from any other lung disease. Dysentery was the cause in 5 cases and enteric fever in 3 Of the year's admissions 40 owed their insanity to one or other of the various moral causes always found in asylum statistics such as domestic trouble and mental anxiety. Intemperance in drink was the cause in 23 cases, hereditary influence in 44, previous attacks in 35, and 31 were of unknown origin. Dr. White says that in former years he attributed "the sporadic cases of dysentery and enteric fever to structural and sanitary defects, and he believed that with the remodelling of the institution, plastering the walls, and renovation of the floors, they would disappear." This opinion was not borne out by subsequent events, and in September last an epidemic of enteric broke out, which Dr. White traced to the water; and the alteration in the supply of water was followed by the cessation of the epidemic. As regards the dysentery also there has been so much diminution in the number of cases that Dr. White could on the date of his report say the asylum was almost free from it, and he adds that he is "more than ever convinced that the predisposing cause of asylum dysentery is chronic constipation in patients of low nerve force." There may be something in this theory, but it does not account for the fact that the disease often absents itself for many months, and then mysteriously returns. Still Dr. White's opinion must carry weight, and it would be a good thing if he and his fellow medical superintendents would investigate the point. The chaplain received a pension of £150 a year, and the chief nurse one of £112. The health of the latter had broken down under the strain of her work, and it is to be hoped that freedom from this will conduce to her recovery. We congratulate Dr. White and also the senior assistant medical officer on their increases of salary.

DERBY BOROUGH ASYLUM.

On January 1st this asylum contained 325 patients, and at the close of the year the number had fallen to 312. There were 71 first admissions, 15 readmissions, 39 recoveries, 5 discharged relieved, and 37 died. The total number under treatment was 411, and the average number resident was 323 The recoveries stand at the very high percentage of 50 on the admissions, and the deaths stand at 11·4 per cent. of the average number resident, which is also high. Of the year's deaths 16 were caused by cerebral and spinal diseases, 1 by consumption, and 2 by pneumonia. Of the year's admissions 15 owed their insanity to various moral causes, and physical causes alone or in conjunction account for the remainder save 1, in whom no cause could be ascertained. Of these physical causes heredity comes first with 29, previous attacks next with 20, and drink with 17. Dr. Macphail says that the average period of residence in those patients who recover is longer than it formerly was, not only in his own asylum, but in others, and he says that "the only explanation seems to be that an appreciable change is gradually taking place in the type of cases of mental disease. Not only are the mental symptoms more severe and prolonged, but the general vitality of the patients is lower." These are most interesting, if unpalatable facts, and we should like to see what bearing the statistics of a few asylums taken collectively would have on them. In spite of the fact that a villa has been built and opened for the reception of patients, the female side of the asylum has been nearly full, and previous to the opening of that annexe it was somewhat overcrowded. The committee record "their appreciation of the highly satisfactory manner in which Dr. Macphail continues to discharge the responsible and onerous duties which devolve upon him." The weekly charge for borough patients is 11s. 1d. per head, and that for out-borough cases is 14s., while private patients pay from 17s. 6d. to 21s. The charges for private patients seem fair and remunerative, but they should never go beyond the latter sum, otherwise the borough would enter into competition with private asylums which should neither be the object of admitting private cases or the result of it.

SUNDERLAND BOROUGH ASYLUM.

On the first of January this asylum contained 327 patients and at the end of the year the number had risen to 343. There were 94 first admissions, 20 readmissions, 49 discharged recovered, 11 discharged relieved, and 37 deaths. The total number under treament during the year was 438, and the average number resident was 335. Calculated on the admissions the percentage of recoveries is 44 1 and this, though lower than in the previous year, is still above the average in English asylums. Calculated on the average number resident the deaths stand at 11 per cent. which is rather higher than usual. Of the year's deaths 15 were caused by cerebral and spinal diseases, 5 by consumption, 7 by pneumonia, and 2 by colitis. Of the year's admissions 15 were supposed to owe their insanity to domestic trouble, adverse circumstances, and mental anxiety. Intemperance in drink accounts for 28, hereditary influence for 38, and puberty and adolescence are credited with 22.

ract-

When speaking of the causes of insanity and of the high number of admissions during the year, Dr. Middlemass shows that when the South African war was at its period of greatest national stress there was a marked diminution of the occurrence of insanity throughout the whole country, and that since the end of the war there has been a decided increase. While admitting that the subject is a complex one, Dr. Middlemass thinks that the sobering effects of adversity, if not overwhelming, may have some influence in checking not only insanity, but crime of various kinds. Of course, this is possible, even probable, but one would hardly know how to set about its proof. When Dr. Middlemass touches on the great question of wages he is on much firmer ground, for it would seem to be quite certain that "when wages are high crime, drunkenness, and insanity are also high, and that the converse is equally true." The cost per head per week is 11s. 8¾d.

KIRKLANDS ASYLUM FOR LANARK AND GOVAN.

There were 205 patients in this asylum at the beginning of the year, and 211 at the end of the year. The first admissions numbered 84; the readmissions 10; the recovered 35; the discharged relieved 20, and the deaths 24. The total number under treatment during the year was 295, and the average number resident was 213. Calculated on the admissions the recoveries stand at 37·2 per cent.; and calculated on the average number resident the deaths stand at 8 per cent. Of the year's deaths 16 were caused by cerebral and spinal diseases, and only one by consumption. Domestic worry and religion were the assigned causes of insanity in six of the admissions; intemperance in drink in 11; hereditary influence in three, and in 35 no cause was known. With such a high proportion of "unknowns" the table is not of much use; but as Dr. Skeen truly says: "the causes of insanity are invariably difficult to trace, at least with any accuracy, due either to a deliberate intention to mislead, or to a want of proper knowledge on the part of relations and friends"; and when referring to alcoholism as an assigned cause Dr. Skeen observes that "many cases are sent on to us as due to alcoholism, whereas in reality the alcoholic habit has probably been due to mental derangement." The rate of maintenance for district patients is 9s. 6d. a week; and out district cases are charged 12s. 4d.

